# Measurement of diamine oxidase (DAO) during low-histamine or ordinary diet in patients with histamine intolerance

**DOI:** 10.1038/s41430-024-01448-2

**Published:** 2024-05-20

**Authors:** Georgios Rentzos, Adina Weisheit, Linda Ekerljung, Jenny van Odijk

**Affiliations:** 1 Department of Internal Medicine and Clinical Nutrition, Institute of Medicine, Sahlgrenska Academy, University of Gothenburg, 405 30 Gothenburg, Sweden; 2 Department of Respiratory Medicine and Allergology, Sahlgrenska University Hospital, 413 46 Gothenburg, Sweden; 3 Krefting Research Centre, Institute of Medicine, University of Gothenburg, 405 30 Gothenburg, Sweden

**Keywords:** Randomized controlled trials, Signs and symptoms

## Abstract

**Background/Objectives:**

Quantification of diamine oxidase (DAO) concentrations in serum has been proposed as an adjunctive diagnostic modality for the evaluation of histamine intolerance (HIT). Limited empirical data exist concerning the influence of dietary patterns on DAO levels.

**Subjects/Methods:**

In the context of a prospective study employing a crossover design, 18 individuals diagnosed with HIT were randomized to initiate either a low histamine diet (LHD) or a conventional mixed diet (MXD). Serum DAO concentrations were measured at the commencement of the study and following each dietary phase. A control group underwent analogous DAO assessments without imposition of dietary constraints.

**Results:**

During the time when a diet restricted in histamine was implemented, noticeable differences in changes in DAO levels did not become apparent when compared to the changes observed during the mixed (MXD) phase. Specifically, among the group, 10 of the 18 patients exhibited elevated DAO values subsequent to the LHD regimen, while the remaining eight displayed either reduced or unchanging DAO levels. The prevalence of elevated DAO levels in the LHD group did not differ significantly from that observed in the control group during the MXD phase. Additionally, during the LHD phase, patients reported a significant reduction in gastrointestinal and cutaneous symptoms.

**Conclusions:**

This prospective investigation underscores the enduring utility of a histamine-restricted diet, coupled with structured dietary reintroduction, as an efficacious diagnostic approach for individuals presenting with suspected food-related histamine hypersensitivity. Notably, the measurement of DAO levels appears to furnish only a limited capacity to discern dietary-induced fluctuations. Notwithstanding, the dynamics of DAO alteration do not appear to exhibit a discernible association with specific dietary patterns, a finding consistent across both patient and control groups.

## Introduction

Histamine is a bioactive amine that is synthesized through the decarboxylation process of its precursor, histidine, which is an essential amino acid [1]. The European Food Safety Authority (EFSA) states that bioactive amines presented in foods can pose a health risk to consumers [1]. In the context of histamine, it is commonly associated with adverse reactions categorized as non-allergic food hypersensitivity, as classified by the World Allergy Organization [2]. Poisoning due to ingested histamine is considered to be a considerable histamine-related toxic reaction [3]. The prevalence of histamine intolerance (HIT) as a non-IgE-mediated subtype of hypersensitivity is currently unknown, but it is estimated to affect approximately 1% of the general population, with a higher incidence among middle-aged individuals [4]. Nevertheless, it is important to note that the actual impact of histamine intolerance could be more substantial than the current estimations suggested [5]. Over the past decade, HIT has gathered increased attention both socially and scientifically, resulting in a significant rise in publications in this field [6].

Histamine is found in various foods such as meat, cheese, fish, and alcoholic beverages. The concentration of histamine content in these foods varies, as it increases with maturation and bacterial contamination [7]. Therefore, determining the amine content in specific foods is important since the concentration may differ between different products [4]. The impact of combining foods with varying concentrations of different amines in patients with histamine hypersensitivity is currently unknown. However, a recently published in vitro study has demonstrated that other biogenic amines can interfere with histamine metabolization by diamine oxidase (DAO) [8].

Histamine, whether it is arising endogenously or exogenously from food, is metabolized through two known degradation pathways: methylation by Histamine-N-methyltransferase (HNMT) and oxidative deamination by diamine oxidase (DAO) [6]. DAO activity can be measured in serum samples, intestinal mucosa, and in stool samples [9, 10]. The determination of DAO levels and the interpretation of results have been contradictory in some published studies, leading to ongoing interest in studying whether symptoms correlate with the measured DAO levels [11–14]. In a study of adult patients with symptoms related to histamine intolerance, the DAO levels in the blood were significantly lower compared to a control group [11]. A small retrospective study found lower DAO levels in the blood of histamine intolerant subjects compared to healthy volunteers [13]. Another retrospective study concluded that the diagnosis of histamine intolerance could be based on low DAO levels and more severe symptoms, although this study lacked a control group [12]. Another study with a retrospective design based on both patients and a control group could link DAO levels to the probability of a diagnosis of histamine intolerance [14].

The positive effect of a histamine-reduced diet on patients with chronic spontaneous urticaria but also in patients with chronic eczema and asthma has been sporadically reported previously in some short studies [15–19]. The influence of a histamine-reduced diet on serum DAO levels has been studied to a limited extent. However, in patients with chronic idiopathic urticaria higher histamine plasma levels were measured during the histamine-free diet [20]. Short-term effects on DAO levels could not be detected during a challenge with a histamine-rich meal in patients compared to healthy controls [21]. Additionally, the daily profiling of DAO serum activity showed reduced levels during the daytime in a subgroup of patients with histamine intolerance compared to patients with IgE-mediated food allergies, although the DAO levels were not measured during any amine-free dietary interventions [22]. In another study comprising a group of patients with low DAO levels and retrospectively collected information on diet adherence and related symptoms, an increase in serum DAO levels among patients who strictly followed a diet was found. An increase in DAO levels was also observed in the group without a specific diet regimen [23].

More data is needed to understand how DAO activity levels are affected by an ordinary mixed diet and a diet with low histamine content. The aim of this study was to assess DAO activity measured in serum in relation to diet (mixed compared to a low histamine diet) and to evaluate self-assessed food-related symptoms associated with histamine hypersensitivity during the dietary intervention period.

## Methods

The study was conducted as a randomized controlled trial using a crossover design. A total of 20 individuals over the age of 18 were referred to the Department of Allergology at Sahlgrenska University Hospital in Gothenburg for investigation of suspected HIT. Between 2018 and 2022, a total of 27 participants were eligible for inclusion in the study. All patients had been diagnosed with histamine intolerance by allergologist physician according to the current guidelines [6, 10]. IgE-mediated food allergy was excluded. The exclusion criteria for study invitation included adherence to a vegan or incompatible diet with the study protocol and diagnosed inflammatory or systemic rheumatologic disease.

The participants were requested to complete a questionnaire regarding atopic diseases, gastrointestinal symptoms, medications, and menstrual cycle status (if applicable). A questionnaire developed and used in an earlier study [24] was adapted for this purpose.

**Fig. 1:**
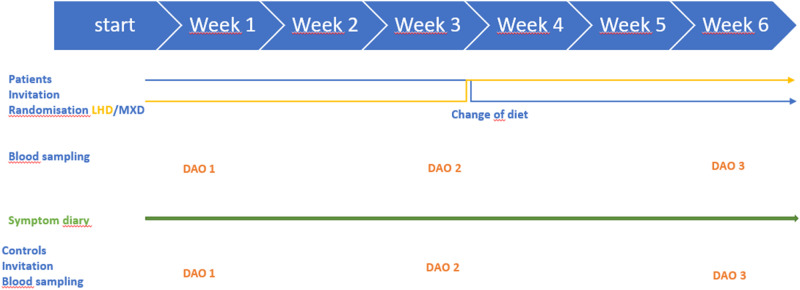
Setup of the study. Patients were randomly assigned to undergo two different 3-week dietary interventions: a low histamine diet (LHD) and a regular mixed diet (MXD). Blood samples were collected at three specific time points for measuring the levels of diamine oxidase (DAO) in both groups. The first sampling (DAO 1) occurred before the dietary interventions started, the second after three weeks of adhering to the mixed diet (DAO 2), and the third after three weeks of switching between the low histamine and mixed diets (DAO 3). Throughout the entire six-week dietary intervention, patients diligently recorded their symptoms in designated diaries. The control group did not undergo any dietary interventions, but blood samples for DAO quantification were collected at the same three-time points as in the patient group.

The participants were randomly assigned to start with either a low histamine diet (LHD) or an ordinary mixed diet (MXD) with a simple randomisation model (Figure 1) and were instructed to follow the assigned diet for a duration of 3 weeks. The LHD involved the reduction of foods and beverages known to be high in histamine content, as determined by the European Food Safety Authority (EFSA) report and implemented by the Swedish Food Agency [7]. During the mixed diet (MXD) phase, patients were advised to consume at least two food items per day that contained higher amounts of histamine (at least one portion of matured cheese, charcuterie, canned or smoked fish, shellfish, fermented products) and otherwise to adhere to a mixed diet regime. Throughout the study, all patients maintained regular contact with the study dietitian during the 3-week periods of dietary interventions to ensure adherence to the diet and protocol.

Participants recorded various food-related symptoms in a symptom diary using a previously validated scale from another study [25]. Symptoms were rated on a scale of 0 to 3, with 0 indicating no symptoms and 3 indicating severe symptoms. The recorded symptom categories included mainly gastrointestinal pain (as well as altered stool consistency such as loose stool or diarrhoea sporadically in a few individuals), skin symptoms (flush, itching, rash, urticaria), and headache severity. Participants were allowed to manually add any additional symptoms not covered by the symptom diary in open questions.

Measurement of diamine oxidase (DAO) activity in serum was performed at the beginning of the study, after 3 weeks of diet intervention, and again after another 3 weeks (Figure 1). Patients were instructed not to use additional medication with antihistamines, glucocorticoids or anti-inflammatory drugs four weeks prior and under the dietary intervention. Additionally, the use of DAO enzyme supplements during the study period was not permitted and the participants were not under any medication with drugs that could block the DAO activity reported in the literature [26, 27].

The control group, consisting of 9 subjects without any reported histamine-related food hypersensitivity, underwent the same procedure with analysis of the DAO levels simultaneously as the patient group during the same 3-weeks intervals during the dietary interventions and were asked to complete the same questionnaires. No dietary restrictions were imposed on the control group, and they were instructed to maintain their regular diet during the entire study period.

### Measurement of DAO in serum

The measurement of DAO levels in the samples was performed using a commercially available kit (DAO-REA Sciotec, HS 421-37; Tulln an der Donau, Austria) according to the manufacturer’s instructions [28]. According to levels defined by the assay manufacturer, values < 3 U/mL were considered to be distinctively decreased levels of DAO, 3–10 U/mL were considered to be slightly decreased and ≥10 U/mL was considered as normal levels.

### Statistics

Statistical analyses were conducted using SAS 9.4 from SAS Institute Inc., Cary. For continuous variables of DAO, median and minimum-maximum values are presented. DAO values were log-transformed to reduce skewness. Categorical variables are expressed as numbers. Differences between groups were assessed using Friedman’s test and GLM/Mixed models. Symptom analysis and corresponding box plots were generated using R Studio Version 1.3.959. The analysis was based on aggregated symptom data, where daily values for each symptom (stomach pain, skin symptoms, headache) were summed for each diet (low histamine diet and mixed diet) to yield a single value per diet per individual. Daily data values for the outcome “stools” were similarly summed for each symptom and diet, and comparisons were made using a paired t-test. Statistical significance was set at *p* < 0.05 for all comparisons.

The study was approved by the Regional Ethics Board in Gothenburg, Sweden (Document no. T351-17).

## Results

During the observational period spanning from 2018 to 2022, a total of 27 patients met the eligibility criteria for participation, and subsequently, 20 patients consented to participate in the study. Notably, two patients withdrew from the study for distinct reasons: one due to intolerable adverse reactions encountered during the mixed diet (MXD) phase and another due to unrelated issues.

Within the patient cohort, a subgroup of 12 out of 18 individuals presented with atopic conditions, encompassing six cases of allergic rhinoconjunctivitis. Among this subgroup, three cases featured optimally managed asthma, and one case presented with atopic eczema, while the remaining six patients exhibited no concurrent atopic conditions. Importantly, none of the recruited patients had pre-existing gastrointestinal ailments, such as irritable bowel disease, celiac disease, non-celiac gluten sensitivity (NCGS), carbohydrate or lactose intolerance, inflammatory bowel disease, eosinophilic disorders, or systemic conditions including mast cell disorders or rheumatic disorders. Furthermore, none of the female patients in the study were pregnant.

Among the ten participants initially recruited for the control group, nine individuals met the inclusion criteria. Four of these individuals had allergic rhinoconjunctivitis but did not possess a medical history or clinical manifestations indicative of oral allergy syndrome (OAS) or food-related symptoms. Crucially, none of the control group subjects reported experiencing food-related hypersensitivity or associated symptoms (Table 1a).

**Table 1:** a: Descriptive data of patients and controls which participated in the study, b: DAO values in the serum in both patients and controls during the different dietary interventions.

a		
	Patients *n*: 18	Controls *n*: 9
Age, median (range)	39 (26–69)	51 (22–53)
Gender, female/male (*n*)	14/4	7/2
Any atopy reported (*n*)	12	4
GI symptoms reported (*n*)	12	0
Asthma diagnosed (*n*)	3	0

b			
During dietary intervention	Patients *n*:18	During no dietary intervention	Controls *n*: 9*
Baseline, Median (range)	9.8 (2.0–37.0)	Baseline	12.3 (7.5–27.1)
Week 3: After LHD diet	8.7 (2.3–42.0)	Week 3: DAO 2	13.3 (7.8–26.3)
Week 6: After MXD diet	9.1 (2.7–50.0)	Week 6: DAO 3	15.0 (7.6–26.3)

*Mixed diet for whole period.

*LHD* Low histamine diet.

*MXD* Mixed diet.

*DAO* Diamine oxidase.

### DAO levels in relation to diet

**Fig. 2:**
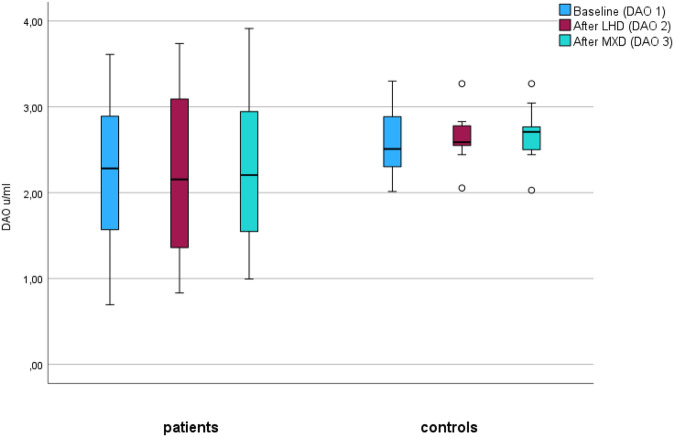
Patient group. Analysis of the diamine oxidase (DAO) values in patients during three phases of a crossover dietary intervention - baseline, Low Histamine Diet (LHD), and Mixed Diet (MXD). **Control group:** Analysis of the DAO values in a control group without dietary restrictions. No significant fluctuations or differences in DAO levels were observed either between patients and the control group or among the different dietary phases within the patient group (*p* > 0.05). This assessment was performed using the non-parametric Friedman’s ANOVA test.

The median baseline diamine oxidase (DAO) activity was 9.8 U/ml within the patient group and 12.3 U/ml within the control group (as presented in Table 1b). It is noteworthy that the intergroup disparity in DAO activity at baseline did not attain statistical significance (Table 1b). An inclination toward elevated DAO activity levels following the low histamine diet (LHD) regimen was observed in 8 of the 18 patients, while the remaining 10 patients exhibited stable or diminished DAO levels after a 3-week period of LHD. Further analysis of DAO levels, encompassing assessments conducted prior to and during the dietary intervention under a crossover design (baseline, post-LHD, and post-mixed diet phases), revealed no statistically significant differences in DAO levels across distinct phases and dietary interventions, as ascertained through non-parametric testing employing Friedman’s analysis of variance (ANOVA), as illustrated in Figure 2.

### Symptoms in relation to dietary regime in patients and controls

**Fig. 3:**
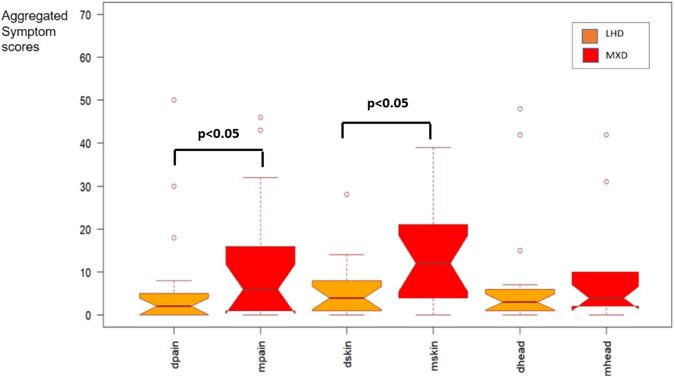
Symptom score and the various symptoms reported by the patient groups while following the Low Histamine Diet (LHD), specifically gastrointestinal pain (dpain), skin-related symptoms (dskin), and headaches (dhead), compared to the symptom scores during the Mixed Diet (MXD), including pain (mpain), skin-related issues (mskin), and headaches (mhead). Symptoms from the gastrointestinal tract (mpain) and skin (mskin) were found significant higher (*p* < 0.05) during the MXD compared to LHD. Aggregated symptom scores are defined whereas daily symptom values for an individual are added for a particular symptom (gastrointestinal pain, skin symptoms, headache) and a diet (LHD respectively MXD) to provide one single aggregate value for that symptom per individual and per diet.

To assess the symptoms related to intake of histamine, daily data values for each symptom category (gastrointestinal pain, skin, headache) were aggregated for each diet (LHD and MXD) to generate a total symptom score per diet and per individual. These scores were compared using a paired *t*-test. The analyses revealed significant differences in the symptom categories, specifically in gastrointestinal pain, but even in skin symptoms, as well as in the total score for all symptoms between the LHD diet and the mixed diet. However, no significant differences were observed concerning headache severity during LHD compared to MXD (Fig. 3). The control group did not report any symptoms during the continued ordinary diet (data not shown).

## Discussion

DAO has been subjected to analysis as a diagnostic modality for histamine intolerance, as verified by various studies [11–13, 23]. Nevertheless, divergent guidelines and uncertainties concerning its diagnostic efficacy have been prominently raised [9, 10, 21, 29]. Prior research has suggested that long-term histamine elimination diets are associated with the recovery of DAO levels in individuals reporting histamine intolerance [23]. In our prospective study, no significant difference in DAO activity was observed based on the type of diet. Baseline DAO values exhibited a wide distribution, with approximately half of the participants having values below the suggested cut-off of 10 U/ml. Baseline DAO values in the control group did not differ significantly from those in the patient group, indicating that DAO cannot be utilized as a diagnostic tool.

When comparing DAO levels between different diets using a crossover interventional model, our findings indicate that the LHD applied for three weeks had no impact on the DAO levels, nor did the MXD. These results contradict the findings of a study conducted by Lackner et al., which employed a similar diet model [23].

The analysis of subjectively self-reported symptoms throughout the study period revealed that patients experienced significantly more skin symptoms (flushing, itching, urticaria) and gastrointestinal pain when consuming an MXD which is in accordance with previous observations and publications [4, 30]. However, no increase in the occurrence of headaches or other neurological symptoms was observed when shifting from LHD to MXD with the content of foods rich in histamine.

The histamine-restricted diet appears to exhibit a positive impact on various chronic symptoms, some of which may be of a food-related nature. Previous observations have demonstrated a prominent amelioration of cutaneous manifestations in certain patients with atopic dermatitis following adherence to a diet with low histamine content [31]. Additionally, the literature indicates an association between the consumption of histamine-rich foods and refractory chronic urticaria, albeit with concurrent pharmacological relief and improvement in urticaria symptoms during LHD [32]. Interestingly, it has been observed that individuals with chronic food-related gastrointestinal symptoms, carbohydrate malabsorption, and non-celiac gluten enteropathy may benefit from reduced histamine intake [33]. This suggests a plausible link between the ingestion of elevated histamine levels in foods and the manifestation of localized intestinal enteropathy in the gastrointestinal tract, of non-immunologic origin [34].

In light of these findings, it is reasonable to assert that a diagnostic exploration of a dietary intervention with low histamine content should be considered for all patients presenting with chronic diffuse symptoms, particularly when clinical history suggests a potential association with food-related etiology. Additional research is needed to establish the optimal extension of a low-histamine diet (LHD) due to the fact that specific foods contain varying levels of histamine and even other biogenic amines within the same food items [35]. It is important to note that the current commercial technique for analyzing Diamine Oxidase (DAO) from Sciotec (DAO-REA 3H) employs putrescine as a substrate. The choice of DAO substrate, whether putrescine or the native substrate histamine, can affect the analysis in various ways. In Sweden, the Sciotec method is the only available approach for determining DAO levels. Consequently, in future studies, it may pose a challenge to ascertain whether the determination of DAO levels would exhibit variations when utilizing a method that exclusively employs the original histamine substrate. This study stands out as one of the few prospective studies utilizing a crossover design, with continuous diet monitoring conducted by a dietitian to ensure adherence and collect reliable data. The study’s limitations include small sample size and challenges in participant recruitment, particularly exacerbated during the COVID-19 pandemic when adhering to a crossover diet proved demanding. Notably, a few participants withdrew due to their reluctance to revert to the standard mixed diet after experiencing significant symptom alleviation through the histamine-reduced diet. Nevertheless, it is important to highlight that these patients maintained close contact with the study coordinator throughout various diet periods, ensuring meticulous adherence to the diverse diets tested during the study duration. Furthermore, it is desirable to subject the control group to the same dietary intervention as the patient group. However, participants without any food-related issues showed limited motivation.

In conclusion, this cross-sectional study demonstrates that the histamine-reduced diet remains the only reliable and eligible diagnostic tool for patients with suspected non-IgE mediated food-related histamine intolerance, while the analysis of the DAO levels showed to be inconclusive as a diagnostic tool. Changes in DAO did not seem to be related to the type of diet in patients. No variation in the DAO levels could be demonstrated in the control group despite a constant diet. In this context, larger prospective studies with more motivated participants are necessary to further explore the potential utility of measuring DAO levels in this specific patient population.

## Data Availability

The data that supports the findings of this study are available upon reasonable request from the corresponding author (JvO). The data are not publicly available due to local legislation related to data derived from patients´ medical records.
